# Agenesis of the right lobe of the liver: a case report

**DOI:** 10.1186/s13256-023-04328-8

**Published:** 2024-01-16

**Authors:** Ashenafi Amsalu Feleke, Friehiwot Amsalu Birhanu, Fraol Worku Tirfe, Yohannis Derbew Molla

**Affiliations:** Department of Surgery, College of Medicine and Health Sciences, University of Gondar, Gondar, Ethiopia

**Keywords:** Agenesis, Cholelithiasis, Right hepatic lobe

## Abstract

**Introduction:**

Agenesis of the right hepatic lobe is a rare congenital anomaly. Developmental anomalies of the right lobe of the liver were first reported in 1870 by Heller [6]. Anatomical variations of the liver are common, occur during the normal development of the organ, and correspond to variations in the distribution of liver territories. Agenesis of the right lobe of the liver is an incidental finding revealed by the popularization of ultrasonography, computed tomography, or magnetic resonance imaging due to the condition being asymptomatic.

**Case presentation:**

A 35-year-old Ethiopian female patient presented to the outpatient clinic with a complaint of right upper abdominal pain for 1 year, along with symptoms of fatty meal intolerance and long-standing epigastric burning pain. Her examination was unremarkable, and imaging investigations were suggestive of right hepatic lobe agenesis with multiple gallstones. Therefore, the patient was operated on and discharged with no perioperative complications.

**Conclusion:**

Surgical understanding of such anatomical anomalies is necessary for surgical planning, the appropriate interpretation of intraoperative surgical findings, and the design of postoperative therapy. Here we report a case of right hepatic lobe agenesis with cholelithiasis and cholidocholithiasis and a brief review of right lobe agenesis in the literature.

## Introduction

Agenesis of right lobe of liver is defined as the absence of liver tissue lateral to the main lobar fissure on the right side with preservation of middle hepatic vein without previous disease or surgery [1]. Agenesis of right lobe of liver is an extremely rare congenital anomaly [2]. Nevertheless, they have been known to anatomists for quite some time. In the past, these anomalies were discovered fortuitously on autopsy or postmortem dissection [3]. Agenesis of the right lobe of the liver is an incidental finding revealed by the popularization of ultrasonography, computed tomography, or magnetic resonance imaging due to the condition being asymptomatic [4]. Agenesis of the main lobe of the liver is an extremely rare congenital anomaly, affecting the left lobe more often than the right. Total agenesis of the liver is incompatible with life. There are only 31 reports of agenesis of the right lobe of the liver in the literature. This anomaly is caused by the developmental failure of the right portal vein or an error of mutual induction between the primitive diaphragm and the endodermal diverticulum representing the primitive liver [3]. Lobar agenesis can be complicated by symptoms of atypical cholecystitis, volvulus of the stomach, or portal hypertension. Portal hypertension is believed to be a result of normal portal venous influx against a reduced intrahepatic vascular bed, while biliary symptoms arise because biliary stasis occurs secondary to compression or torsion of the cystic duct [5].

Generally, it is associated with other anatomical alterations, such as hypertrophy of other liver segments, colonic interposition between the liver and diaphragm, right diaphragmatic hernia, portal hypertension, or an anomalously positioned gallbladder [1]. The gallbladder may be posterior, lateral, or superior to its expected position. All these conditions render cholecystectomy technically challenging, especially under laparoscopy [4]. Surgical understanding of such anatomical anomalies is necessary for surgical planning, the appropriate interpretation of intraoperative surgical findings, and the design of postoperative therapy [6].

## Case presentation

A 35-year-old Ethiopian female patient presented to the outpatient clinic with a complaint of right upper abdominal pain for 1 year along with symptoms of fatty meal intolerance and long-standing epigastric burning pain, but no history of steatorrhea, urine color change, or jaundice, and 15 years ago, the patient had yellowish eye discoloration (jaundice) but no right upper quadrant pain or fever, and was treated with unspecified oral medication at the nearby health center. The patient had no history of previous surgery, trauma, or any risk factors suggesting hydatid disease of the liver. She had no history of diabetes, hypertension, asthma, or any known drug allergy.

On examination, her vital signs were within normal limits. Her body mass index (BMI) was 23 kg/m^2^. At presentation, she had no jaundice, right upper quadrant tenderness, or any palpable abdominal mass. The rest of the examinations were unremarkable. Viral markers and the autoimmune hepatitis antibody spectrum were negative. The laboratory results showed the values of liver function, urinalysis, and hemostatic test were within normal limits. She was investigated for possible gallstone disease, and imaging was carried out. Ultrasonography showed she had multiple stones in the gallbladder and agenesis of the right hepatic lobe with an enlarged left lobe.

**Fig. 1:**
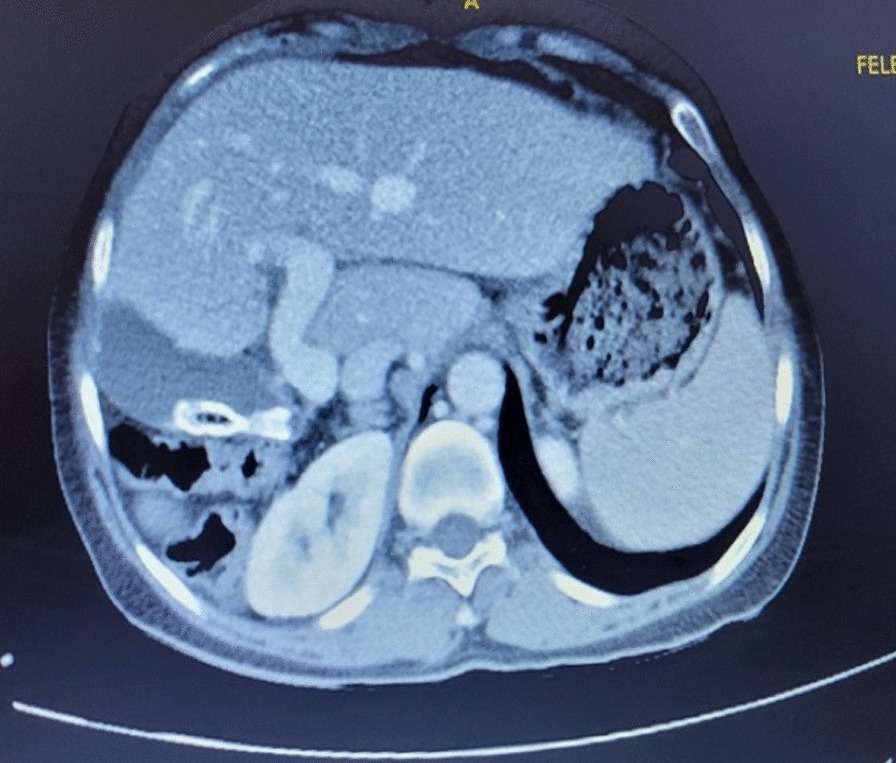
Contrast-enhanced computed tomography scan showing absent right hepatic lobe with compensatory hypertrophy of the left lobe and caudate lobe, absent right portal vein, right hepatic artery, and right hepatic vein

**Fig. 2:**
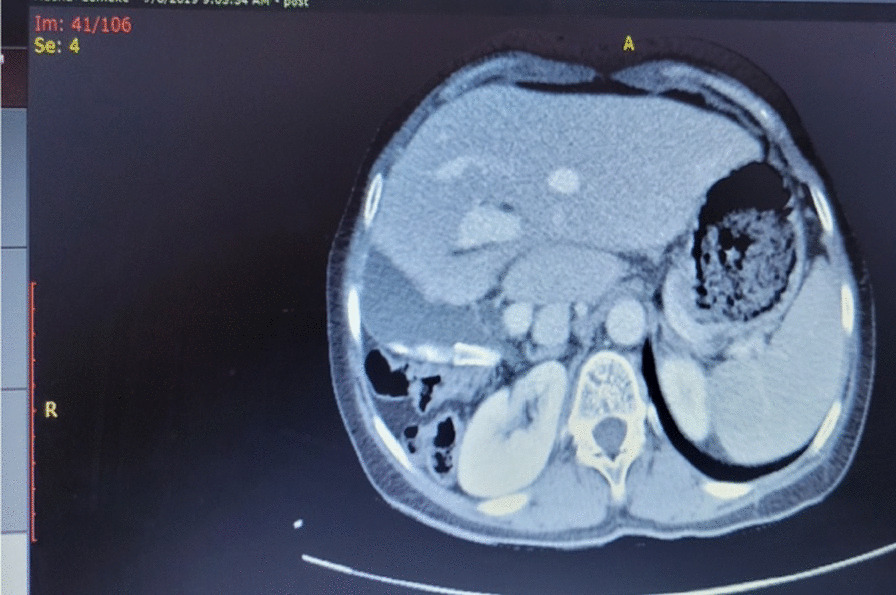
Abdominal computed tomography scan showing retrohepatic gallbladder and multiple radiopaque foci in the gallbladder colonic diaphragmatic interposition

**Fig. 3:**
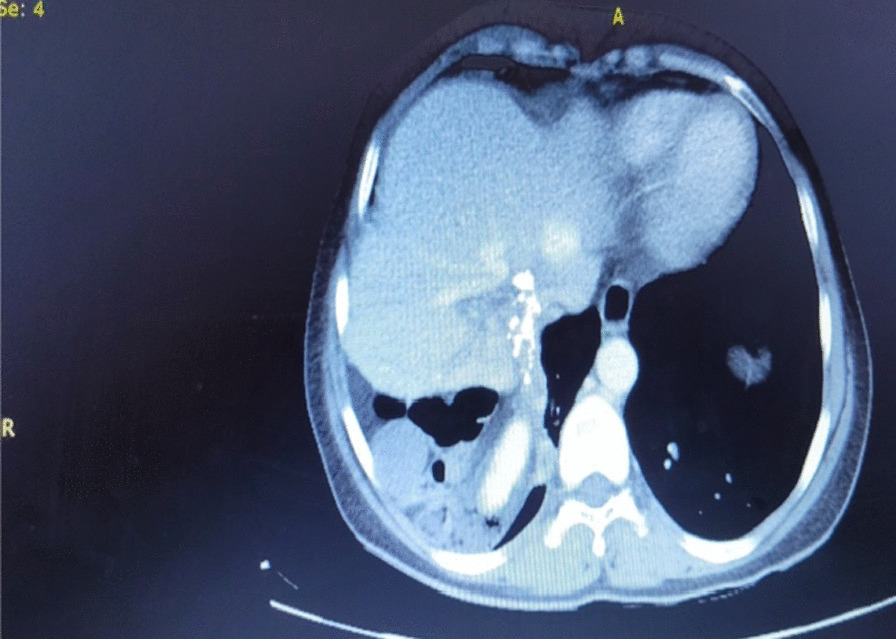
Abdominal computed tomography scan

Computed tomography revealed the absence of the right hepatic lobe with compensatory hypertrophy of the left hepatic lobe and caudate lobe and the absence of the right portal vein, right hepatic artery, and right hepatic vein. Retrohepatic gallbladder and multiple radiopaque foci in the gallbladder and colonic diaphragmatic interposition were also reported. (Figs. 1, 2, 3).

**Fig. 4:**
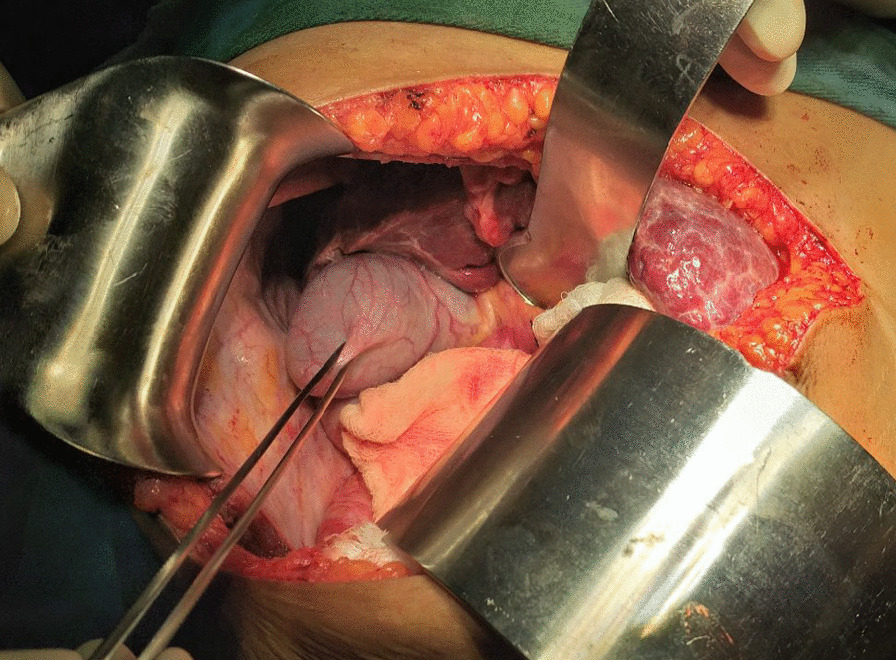
Intraoperative picture showing gallbladder between the liver the diaphragm and have attachment with the diaphragm

**Fig. 5:**
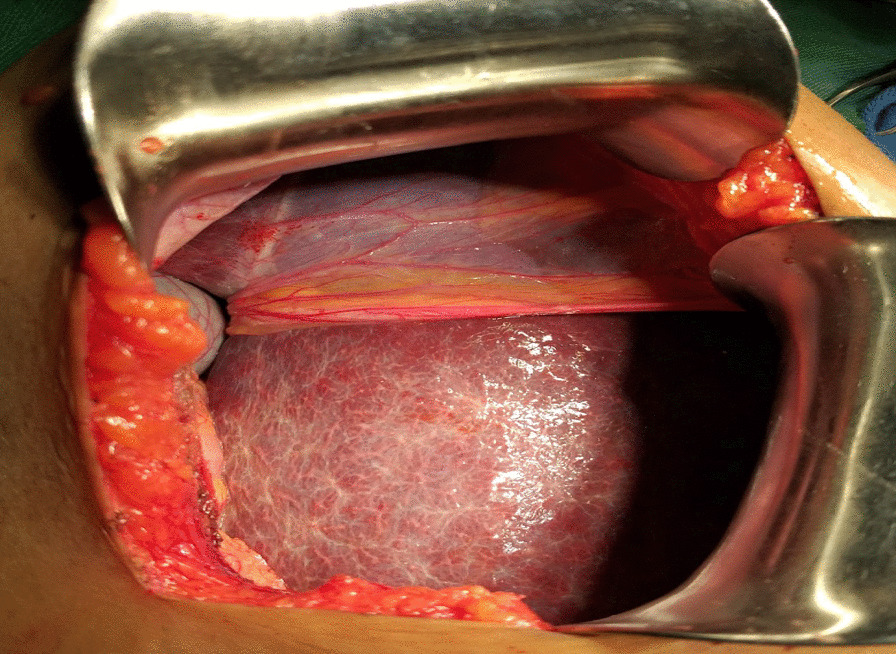
Intraoperative picture showing enlarged left lobe and absent right lobe

**Fig. 6:**
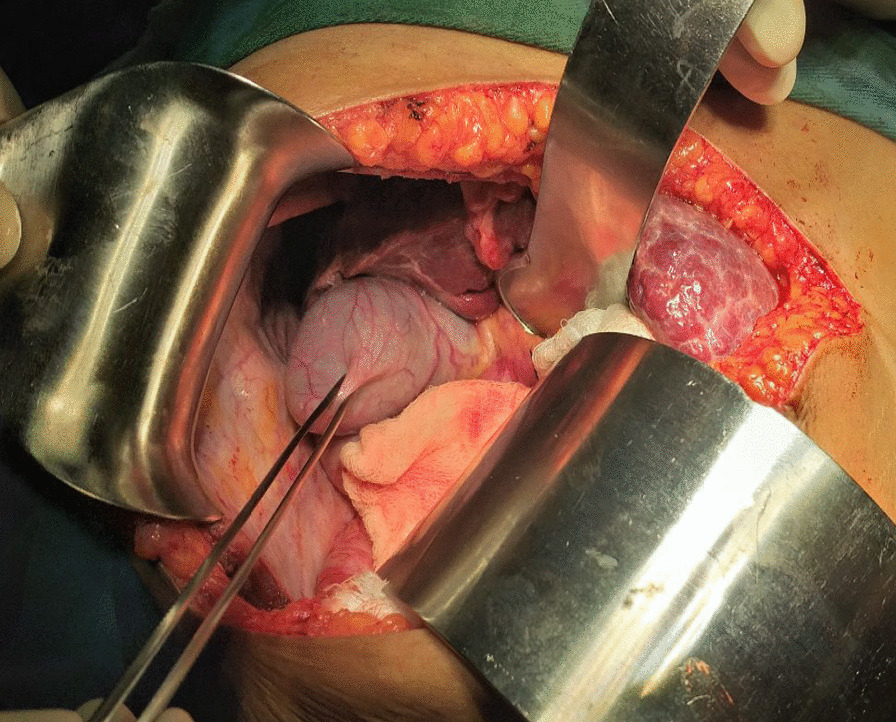
Intraoperative picture showing attachment of gallbladder with the diaphragm

The patient then underwent an explorative laparotomy with a right upper quadrant subcostal incision. The patient was operated on under general anesthesia by a hepatobiliary surgeon and general surgery residents. The findings are as detailed here. We observed a complete absence of the right lobe of the liver with compensatory hypertrophy of the left and caudate lobes and some degree of early cirrhosis. The gall bladder was located posterior to the left lobe of the liver in a vertical position with attachment to the diaphragm and a high-positioned hepatic flexure of the transverse colon. The cystic duct has a tortuous course to enter the “common hepatic duct.” The gallbladder was full of stones, and the common bile duct had a few stones that were palpable. Then cholecystectomy, common bile duct exploration with T-tube drainage, and discharging the patient without perioperative complications were carried out. (Figs. 4, 5 and 6).

**Fig. 7:**
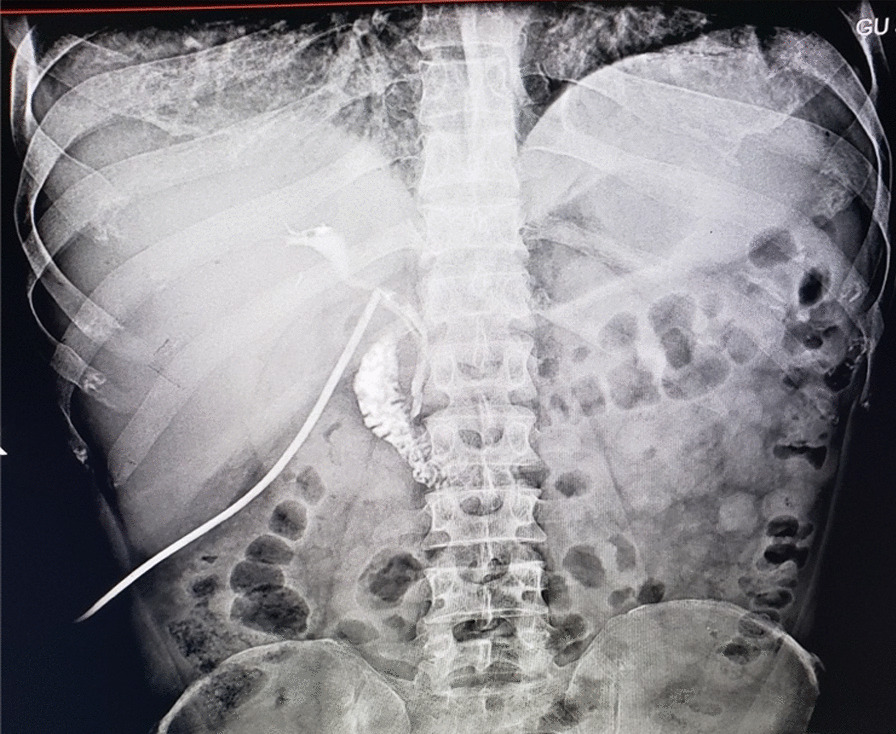
T-tube cholangiogram on the seventh postoperative day showing patent common bile duct with no filling defect

The patient’s postoperative course was smooth. She underwent T-tube cholangiography on the seventh day, which had no retained stones (Fig. 7). She was discharged on the tenth day after all the tubes were removed. Currently, patient comes every 6 months to our outpatient department for checkups and to monitor the progress of the cirrhosis.

## Discussion

The liver primordium appeared in the middle of the third week as an outgrowth of the endodermal epithelium at the distal end of the foregut. This outgrowth, the hepatic diverticulum, or liver bud, consists of rapidly proliferating cells that penetrate the septum transversum, that is, the mesodermal plate between the pericardial cavity and the stalk of the yolk sac. A small ventral outgrowth is formed by the bile duct, and this outgrowth gives rise to the gallbladder and the cystic duct [7]. The liver is the largest gland in the body, weighing approximately 1500 g. The liver has a dual blood supply. The portal vein brings 75–80% of the blood to the liver. Arterial blood from the hepatic artery accounts for only 20–25% of the blood. The hepatic veins (right, middle, and left) are formed by the union of collecting veins that in turn drain the central veins of the hepatic parenchyma and open into the IVC just inferior to the diaphragm. The attachment of these veins to the IVC helps hold the liver in position [8].

Agenesis of the right hepatic lobe is a rare congenital anomaly. Developmental anomalies of the right lobe of the liver were first reported in 1870 by Heller [6]. Anatomical variations of the liver are common, occur during the normal development of the organ, and correspond to variations in the distribution of liver territories [5]. Lobar agenesis is defined as the congenital absence of or incomplete development of liver tissue of the right or left hepatic lobe without prior surgery or hepatic disease [2]. However, the agenesis should be differentiated from lobar atrophy of the right hepatic lobe caused by cirrhosis, cholangiocarcinoma, idiopathic portal hypertension, prior hepatic resection, post-traumatic atrophy, biliary obstruction, veno-occlusive disease, hydatid cyst, or anomalies of other biliary tracts [9].

Advances and the increased use of non-invasive imaging techniques such as ultrasonography, computed tomography, and magnetic resonance imaging led to the more frequent detection of such variant anomalies [10]. CT is the best method for diagnosing hepatic lobar agenesis. Classically, the image reveals the absence of a right lobe with left and caudate lobe hypertrophy [10]. We emphasized the importance of visibility of the right portal vein and its branches in the identification of agenesis and the differentiation of this disorder from aplasia or hypoplasia of the right lobe of the liver [6]. Roentgenograms of the portal vein demonstrate that its right branch is either absent or continues directly into a hepatic vein to empty into the inferior vena cava [3]. Agenesis of the right hepatic lobe is always associated with the absence of the right hepatic artery, right hepatic vein, and right portal vein [5]. Simultaneously, the left portal vein exhibited compensatory thickening. Right hepatic artery branch thinning was observed in some patients. Hepatic vein and inferior vena cava hypoplasia could also be found. A reduction in the size of the hepatic inferior vena cava and compensatory widening of the azygos vein, the hemiazygos vein, and the collateral circulation were found in one patient [6]. In our case, the imaging studies revealed the absence of the right hepatic vein, the right hepatic artery, and the right portal vein.

In cases of such congenital liver anomalies, the gallbladder is often placed on the right side of the liver against the diaphragm in a retrohepatic or suprahepatic location [10]. In our case, the gallbladder is also on the right side of the liver, in a vertical position between the right costal arch and the right dome of the diaphragm. In the case of an atypically located gallbladder, the inferior vena cava can be used instead as another landmark. Although the right hepatic lobe is absent, the inferior vena cava keeps its usual position in contact with the posterior visceral surface of the medial segment of the left hepatic lobe [2]. Previous studies have revealed that the congenital agenesis of the liver lobe affects the left lobe more than the right lobe, and the agenesis of the right lobe occurs slightly more often in men [9]. However, in our case, this rare anomaly occurred in the right lobe and was an incidental finding noticed in a female patient with a complaint of right upper quadrant pain (Table 1).

**Table 1:** Reported cases of hepatic agenesis

Authors	Year of publication	Patients clinical presentation	Investigations	Objective of the paper
Masayuki Kanematsu *et al*. [12]	Published in 1991	A 48-year-old man presented with epigastric pain, white watery diarrhea but no jaundice	The laboratory studies showed normal results Computed tomography (CT) revealed an absence of the right lobe and the caudate lobe of the liver with glossy enlarged left hepatic lobe	This paper describes a rare case of agenesis of the right liver lobe, which was diagnosed using computed tomography (CT), liver scintigraphy, and angiography. The radiological findings and differential diagnosis are reviewed
Soo Jeong Lee *et al*. [13]	Published in July 1993	A 36-year-old man presented with colicky right upper quadrant pain	Normal laboratory studies CT scan showed absent right lobe of the liver with displaced gallbladder forming a blunt angle between the long axis of the gallbladder and CBD	This report describes a patient with agenesis of the right liver lobe, emphasizing the rarity of this congenital anomaly and the limited number of reported cases
Karaman *et al*. [2]	Published in 1997	An obese 55-year-old woman was admitted with abdominal pain, nausea, and vomiting	All laboratory tests were normal CT scan showed total agenesis of the right lobe of the liver and compensatory hypertrophy of the left lobe plus hydatid cyst	The paper presents a case report of a rare congenital anomaly called agenesis of the right lobe of the liver, which is usually accompanied by additional anomalies such as a retrohepatically or suprahepatically located gallbladder. The case report includes a patient with agenesis of the right lobe of the liver with Chilaiditi syndrome and a subdiaphragmatic hydatid cyst
Norihiro Sato *et al*. [14]	Published in 1998	An 84-year-old woman presented with vomiting and weight loss	Normal laboratory tests CT scan showed absence of the right lobe of the liver and an enlargement of the left lobe plus gastric cancer	The paper reports a case of congenital agenesis of the right lobe of the liver detected in a patient with gastric cancer. The diagnosis was established using computed tomography, abdominal angiography, and operative findings. The importance of recognizing this condition is stressed, and the clinical presentation, radiographic appearance, and differential diagnosis are also discussed
Geeta Gathwala *et al*. [15]	February 2003	A 10-year-old male child presented with swelling on the left side of abdomen	All laboratory test were within normal range CT scan showed agenesis of the right lobe of liver with portal hypertension	This article reports a case of agenesis of the right lobe of the liver and provides a brief review of the literature, discussing the diagnosis and differential diagnosis of this condition
Antonio Iannelli *et al*. [4]	Published in May 2005	A 70-year-old woman was admitted with abdominal pain in the epigastrium and upper right abdominal quadrant, nausea, and vomiting	The ultrasound (US) revealed gallbladder stones and ruled out other anomalies	This study focuses on the challenges encountered during laparoscopic cholecystectomy in a patient with congenital agenesis of the right liver. It highlights the importance of preoperative planning and awareness of anatomical variations
Lucas Souto Nacif *et al*. [10]	Published in March 2012	A 32-year-old male patient presented with abdominal discomfort and loss of appetite but no jaundice or urine color change	Laboratory tests and tumor markers were all normal CT scan revealed the absence of the right hepatic lobe, hypertrophied left hepatic segments	This article discusses a case of biliary injury after laparoscopic cholecystectomy in a patient with right liver agenesis. It highlights the challenges and considerations in surgical management
Our case		A 35-year-old woman presented with right upper quadrant pain and fatty food intolerance	Her laboratory studies were within the normal range CT scan showed absence of right hepatic lobe with compensatory hypertrophy of the left hepatic lobe and caudate lobe plus gallstone	The abstract of the paper briefly describes the importance of understanding anatomical anomalies for surgical planning and postoperative therapy. It also reports a case of a rare congenital anomaly, right hepatic lobe agenesis, and cholelithiasis in a 35-year-old female patient

Surgical knowledge of such anatomical agenesis is thus necessary for surgical planning, appropriate identification, and intraoperative findings of the postoperative approach to therapy [1]. We share the opinion with Fuertes *et al*. that this entity is not a contraindication for laparoscopic cholecystectomy, but previous knowledge of the condition is indeed appropriate. The gallbladder may require a different placement of laparoscopic instruments and a different strategy [4]. The compensatory hypertrophy of the medial and lateral segments of the left hepatic lobe as well as the caudate lobe can compress the gallbladder dorsal wall and displace its axis from a right-anterior to a right-posterior direction. Structures in the hepatic pedicle also undergo a partial anticlockwise twist, with the main bile duct running almost posteriorly in the hepatic pedicle. The presence of other anomalies such as portal hypertension, partial or complete absence of the right diaphragm, and a choledochal cyst may render the laparoscopic cholecystectomy even more challenging [3].

In patients with a normal-sized left lobe, the hepatic flexure of the colon may be seen just beneath the lateral portion of the diaphragm, similar to the hepatodiaphragmatic interposition of the bowel known as Chilaiditi syndrome. However, this abnormality is not specific to lobar agenesis of the liver. Therefore, we think that Chilaiditi syndrome is not the appropriate term for the hepatodiaphragmatic interposition of the bowel accompanying lobar agenesis of the liver [6].

In agenesis of the right liver lobe, the lateral segment of the left lobe usually undergoes compensatory hypertrophy, whereas the medial segment and the caudate lobe may or may not be enlarged [11]. The vascularity of the caudate lobe is variable and may be derived from the right or main branch of the portal vein, or from both. This explains why the caudate lobe may be either absent or hypertrophied in agenesis of the right lobe [2]. The reason why some of these patients may lack compensatory hypertrophy of the left lobe is uncertain, but it has been suggested that the process responsible for agenesis of the right lobe also involves the left lobe, although to a lesser degree [6].

According to Pages *et al*., anomalies of morphology related to developmental defects can be categorized as follows: agenesis (absence of a lobe that is replaced by fibrous tissue); aplasia (one of the lobes is small and its structure is abnormal, with few hepatic trabeculae, numerous bile ducts, and abnormal blood vessels); or hypoplasia (one of the lobes is small but is normal in structure) [3, 10]. According to this classification, our case would be categorized as agenesis.

In one study, 35% of patients presented with no evident symptoms, and anomalies were found via routine examination, or in some cases, during an examination for a different cause. None of these right hepatic lobe agenesis patients displayed abnormal laboratory results [6].

The main complications of these anomalies are as follows: 1. cholecystitis and cholelithiasis: may be due to abnormal hepatic anatomy and the resultant displacement of the gallbladder that produces compression or torsion of the cystic duct, leading to bile stasis in the gallbladder and, ultimately, calculus formation; 2. choledocholithiasis: may be associated with abnormal development of the gallbladder, resulting in an elongated, tortuous cystic duct and a relatively low position of the common bile duct. These positional changes may cause the cystic duct and the common bile duct to be in parallel, squeezing the common bile duct. Cystic duct stones coupled with repeated inflammation caused by inflammatory factors in the hepatobiliary triangle resulted in poor drainage and stricture of the bile duct, which is essential for the development of choledocholithiasis and congenital bile duct abnormalities, which might also cause choledocholithiasis; 3. portal hypertension: the probable etiology appears to be a reduction in the number of intrahepatic branches of the portal vein that is not compensated by increased density of the left lobe vasculature. An imbalance between portal inflow and the capacity of the portal bed could be an alternative cause of portal hypertension. Possible vascular resistance in the right portal branches might also cause portal hypertension. However, there are reports of patients with no increase in vascularization of the left hepatic lobe and no evidence of portal hypertension; 4. liver infection and abscess: may be associated with repeated stimulation of bile duct stones, cholestasis, and congenital abnormalities of the biliary tract; 5. biliary tract cholangiocarcinoma: the relationship between aplasia or hypoplasia of the right hepatic lobe and malignant biliary tumors remains to be determined. One explanation for this relationship may be that right hepatic lobe hypoplasia may occur in association with compensatory left hepatic lobe hypertrophy as a consequence of portal vein obstruction due to hilar cholangiocarcinoma; 6. bile pneumatosis: might be related to dysfunction of the sphincter of Oddi and congenital dysplasia of the intrahepatic bile duct; 7. Budd–Chiari syndrome and idiopathic retroperitoneal fibrosis, absence of the inferior vena cava; and 8. pulmonary system alterations and the modification of intestinal position and rotation may also occur [1, 5, 10]. Our patient claimed she was satisfied with the care she was provided.

Literature on right liver agenesis is limited, with only a few reported cases. Here is a summary of the available literature after 1990:

## Conclusion

Though rare, congenital anomaly of the right lobe of liver may be encountered, and surgical management requires a thorough knowledge of the anomalous position of structures around the liver. Imaging showing details of the anatomy of liver and biliary structures is very important for preoperative planning. One may need expertise in radiology to guide in the identification of evidence of an anomaly in the hepatobiliary and pancreatic anatomy, and once said anomaly is identified, the next step will be to carefully plan the surgery after obtaining informed consent from the patient at hand. The course of surgery depends upon the intraoperative finding; ours were detailed in the case report and discussion. Most of these patients present some degree of cirrhosis, so regular follow-up with hepatologist and hepatobiliary and pancreatic surgeon is recommended.

## Data Availability

The authors of this manuscript are willing to provide additional information regarding the case report.

## Abbreviations

CT: Computed tomography
IVC: Inferior vena cava
MRI: Magnetic resonance imaging
